# Outcome evaluation of technical strategies on reduction of patient waiting time in the outpatient department at Kilimanjaro Christian Medical Centre—Northern Tanzania

**DOI:** 10.1186/s12913-024-11231-5

**Published:** 2024-07-09

**Authors:** Manasseh J. Mwanswila, Henry A. Mollel, Lawrencia D. Mushi

**Affiliations:** 1 Kilimanjaro Christian Medical Centre, P. O. Box 3010, Moshi, Tanzania; 2 Department of Health Systems Management, School of Public Administration and Management, Mzumbe, P.O. Box 2, Morogoro, Tanzania

**Keywords:** Electronic medical records, Outpatient department, Waiting time, Block appointment, Patient waiting time, Patient

## Abstract

**Background:**

The Tanzania healthcare system is beset by prolonged waiting time in its hospitals particularly in the outpatient departments (OPD). Previous studies conducted at Kilimanjaro Christian Medical Centre (KCMC) revealed that patients typically waited an average of six hours before receiving the services at the OPD making KCMC have the longest waiting time of all the Zonal and National Referral Hospitals. KCMC implemented various interventions from 2016 to 2021 to reduce the waiting time. This study evaluates the outcome of the interventions on waiting time at the OPD.

**Methods:**

This is an analytical cross-sectional mixed method using an explanatory sequential design. The study enrolled 412 patients who completed a structured questionnaire and in-depth interviews (IDI) were conducted among 24 participants (i.e., 12 healthcare providers and 12 patients) from 3rd to 14th July, 2023. Also, a documentary review was conducted to review benchmarks with regards to waiting time. Quantitative data analysis included descriptive statistics, bivariable and multivariable. All statistical tests were conducted at 5% significance level. Thematic analysis was used to analyse qualitative data.

**Results:**

The findings suggest that post-intervention of technical strategies, the overall median OPD waiting time significantly decreased to 3 h 30 min IQR (2.51–4.08), marking a 45% reduction from the previous six-hour wait. Substantial improvements were observed in the waiting time for registration (9 min), payment (10 min), triage (14 min for insured patients), and pharmacy (4 min). Among the implemented strategies, electronic medical records emerged as a significant predictor to reduced waiting time (AOR = 2.08, 95% CI, 1.10–3.94, *p*-value = 0.025). IDI findings suggested a positive shift in patients' perceptions of OPD waiting time. Problems identified that still need addressing include, ineffective implementation of block appointment and extension of clinic days was linked to issues of ownership, organizational culture, insufficient training, and ineffective follow-up. The shared use of central modern diagnostic equipment between inpatient and outpatient services at the radiology department resulted in delays.

**Conclusion:**

The established technical strategies have been effective in reducing waiting time, although further action is needed to attain the global standard of 30 min to 2 h OPD waiting time.

**Supplementary Information:**

The online version contains supplementary material available at 10.1186/s12913-024-11231-5.

## Background

The Tanzanian healthcare system is beset by prolonged waiting times in its hospitals, particularly in the outpatient departments. The reported contributing factors include the increased need for healthcare due to uncontrolled population growth, an inadequate number of medical experts, underdeveloped healthcare systems, and ineffective referral systems [1]. The audit report from the Ministry of Health on the management of referral and emergency healthcare services at zonal and regional referral hospitals showed a high OPD waiting time. Previous studies suggest that the average waiting time at, Muhimbili National Hospital OPD was 4 – 6 h; Muloganzila Zonal Referral Hospital was 3 – 4 h; Bugando Medical Centre was 2.5 h, Mbeya Zonal Hospital was 3 – 4 h and Kilimanjaro Christian Medical Centre (KCMC) was 6 h [1, 2]. According to these data, KCMC has the longest waiting time of any zonal and National referral hospital in Tanzania. In response to the long waiting time, KCMC implemented a series of interventions that were incorporated into the strategic plan from 2016 to 2021. The interventions included the use of a block appointment system, the transition from paper to electronic medical records (EMRs), the extension of clinic days and the acquisition of modern diagnostic equipment.

Effective scheduling is crucial to minimize patient waiting times. Appointment systems should include rules for setting appointments and sequencing patients' arrivals, aligning them with doctors' schedules. Studies have shown that optimizing block appointment scheduling can significantly reduce patient waiting times without increasing physician idle time [3–5]. Effective appointment scheduling has been shown to significantly reduce patient waiting time in outpatient facilities. A study conducted in the USA demonstrated that planning appointment slots can decrease waiting time by as much as 56%.This evidence suggests that optimizing block appointment scheduling is a viable strategy to enhance outpatient efficiency [6]. Another study in Sri Lanka, demonstrated that implementing a well-structured appointment scheduling system could reduce total patient waiting time by over 60%. Therefore, adopting a block appointment system allows for more efficient allocation of resources and scheduling, ultimately enhancing the overall patient experience and optimizing healthcare delivery [7, 8]. In Mozambique they introduced a block appointment scheduling system to evaluate its impact on waiting time. The findings revealed a reduction in waiting time by 1 h and 40 min (100 min) The study concluded that by introducing block appointment scheduling, patient arrivals were distributed more evenly throughout the day, resulting in reduced waiting times [9].

The implementation of electronic medical records (EMRs) has been shown to offer significant advantages in healthcare delivery, particularly in less developed nations. Evidence indicates that EMRs can decrease patient waiting time, lower hospital operating costs and communication between departments; enable doctors to share best practices. Unlike paper-based records, EMRs provide greater flexibility and leverage, enhancing overall healthcare efficiency [10]. Long waiting times in the OPDs are often exacerbated by inefficiencies in managing patient records. A tertiary medical college hospital in Mangalore, Karnataka, evaluated patient waiting and identified disorganized manual files as a primary cause of delays. These findings underscore the disadvantages of paper-based records and suggest that implementing electronic medical records (EMRs) can greatly enhance efficiency [11]. Reducing outpatient waiting times is a critical challenge for healthcare systems. Evidence from a study in Korea demonstrated that implementing EMRs can significantly reduce waiting time by nearly 60% and enhance operational efficiency. [12]. Addressing long waiting time in the OPD is essential for enhancing patient satisfaction and healthcare efficiency. A systematic survey study aimed at utilizing various models to shorten OPD waiting time found that healthcare providers significantly favored electronic medical records (EMRs) over manual records. The primary reasons cited were significant time savings and a consequent reduction in long waiting time.

 [13]. The issue of long waiting time in outpatient departments (OPDs) is a prevalent problem faced by healthcare facilities worldwide. A study conducted in Brazil applied Lean thinking and an action research strategy to address patient flow issues and identify the causes of prolonged waiting time at the OPD. The study's findings highlighted that many hospitals globally are tackling this issue by investing in electronic medical records (EMRs) to transition away from manual medical records. This evidence suggests that implementing technical strategies, such as EMRs, can significantly improve patient flow and reduce waiting times [14].

Extending clinic days throughout the week has been found to be more effective in reducing waiting times than extending clinic hours. Studies have demonstrated substantial reductions in patient waiting times and increased patient satisfaction following the extension of clinic days. In Canada the study found that extending clinic day was more effective in reducing waiting time than extending clinic hours. Extending clinic days resulted in a 26% reduction in average waiting time, whereas extending clinic hours led to a 16% reduction. This research provides valuable insights for healthcare administrators seeking to optimize clinic operations and enhance patient experience [15]. At a tertiary care hospital in Oman the findings revealed a substantial 56% reduction in patient waiting time following the extension of clinic days. Additionally, patient feedback indicated a high level of satisfaction with the extended clinic days, with 97% of patients reporting satisfaction with the service [16]. Extending clinic days throughout the week has demonstrated promising results in a study conducted at a tertiary care hospital in India. The findings revealed a noteworthy 46% reduction in average patient waiting time following the extension of clinic days. This substantial decrease underscores the effectiveness of extending clinic hours in streamlining patient flow and improving efficiency. Consequently, these results provide compelling evidence supporting the rationale for extending clinic days throughout the week as a viable intervention to alleviate patient waiting times and enhance overall healthcare service delivery [17].

Utilizing modern equipment in healthcare settings has shown significant potential in reducing patient waiting times. A study conducted at a tertiary care hospital in Italy evaluated the effectiveness of modern equipment on patient. The findings indicated a notable reduction in patient waiting time, with an average decrease of 14 min per patient following the introduction of modern equipment. These results suggest that integrating modern equipment into can be a highly effective intervention for improving operational efficiency and reducing patient waiting time [18]. Modern equipment can be instrumental in reducing patient waiting times. A tertiary care hospital in Pakistan revealed that one of the primary causes of prolonged waiting time was the lack of adequate examination equipment. By addressing the equipment deficiencies highlighted in the study, healthcare providers can significantly reduce waiting times, thereby improving patient satisfaction and overall efficiency. Therefore, investing in modern equipment is justified as a strategic intervention to enhance patient flow and optimize healthcare service delivery [19–22]. Modern equipment is essential for reducing patient waiting times in healthcare facilities. An audit assessment conducted in zonal hospitals in Tanzania by the Ministry of Health revealed that outdated equipment, such as x-ray machines, significantly contributed to long waiting time. The limited capacity of these machines meant that only a certain number of patients could be attended to each day, and the equipment required rest periods to avoid overheating. These findings underscore the necessity of updating and maintaining modern medical equipment to improve patient throughput and reduce waiting times [2].

In Tanzania the Ministry of Health has not established the gold standard waiting time for patients to wait for services at the OPD [2]. However the United States Institute of Medicine (IOM) has established their gold standard patient waiting time at the OPD which suggests that medical care should be provided to at least 90% of patients no later than 30 min after their scheduled appointment time [23, 24]. The Patient's Charter of UK, has recommended the same standard as the IOM [25]. The absence of a gold standard waiting time carries several significant implications. It results in inconsistent patient experiences with unpredictable waiting time across facilities, leading to frustration and dissatisfaction. Prolonged and varied waiting time can compromise the quality of care, affecting patient outcomes. Inefficient resource allocation becomes a challenge, hampering the ability to determine staffing and infrastructure needs [26]. This lack of a benchmark reduces accountability, and healthcare providers may not be incentivized to improve waiting time. It adversely affects patient satisfaction, the reputation of healthcare providers, and can exacerbate healthcare disparities. [19]. Hence, the findings from this research will provide valuable insights to the hospital management, enabling them to reinforce substantial improvements in patient waiting time and target areas where progress has been limited within the OPD at KCMC.

The objective of this study is to assess the patient waiting time at KCMC after intervention. Thus, the specific objectives were to determine the OPD patient waiting time since the inception of implementation of the interventions and to assess the effect of technical strategies on patient waiting time.

## Design and methods

### Settings

The study was conducted at Kilimanjaro Christian Medical Centre (KCMC) Outpatient department. KCMC is located in the foothills of the snow-capped Mount Kilimanjaro. It is one among the six zonal consultant hospitals in Tanzania. It was established in 1971 as a Zonal Referral Consultant hospital owned by the Evangelical Lutheran Church of Tanzania (ELCT) under the Good Samaritan Foundation (GSF). The referral hospital was established in order to serve the northern, eastern and central zone of Tanzania. Its record in medical services, research, and education has significant influence in Tanzania, East Africa and beyond. It serves a potential catchment population of 15 million people with 630 official bed capacity. The hospital has a number of clinical departments namely, General Surgery, Orthopaedic and Trauma, Dental, Dermatology, Paediatric, Eye, Otorhinolaryngology, Obstetric and Gynaecological and Internal Medicine. There are 1300 staff seeing about 1200 outpatients and 800 inpatients. The hospital has 100 specialists, 52 medical doctors, 465 nurses and the remaining 643 are paramedical and supporting staff. This area was chosen because the outpatient department at KCMC sees a high volume of patients on a regular basis from diverse backgrounds, including rural and urban populations of Tanzania as well as neighbouring countries. For instance in the year 2022, a total of 301,091 patients attended KCMC hospital, of which 92% (*n* = 277,013) attended the OPD. This high patient volume made it a suitable location for studying patient waiting time.

## Study design

This was an outcome evaluation whereby an analytical cross-sectional design was used to examine the subject matter. This study employed a mixed method explanatory sequential evaluation approach.

### Population and sampling

The study surveyed 412 patients quantitatively and conducted qualitative interviews with 12 patients and 12 healthcare providers. In addition patients who were involved in quantitative were not involved in the qualitative sample. The quantitative sample size was obtained using the following formula [27]:

$${\varvec{n}}=\frac{{{\varvec{Z}}}^{2}{\varvec{P}}(1-{\varvec{P}})}{{{\varvec{d}}}^{2}}$$

Whereby: -

*n* = sample size.

Z = is the standard normal deviation which is 1.96 for a 95% confidence interval.

P = is the percentage of patients attending the OPD at KCMC is estimated to be 0.5, attributed to the absence of prior research data.

d = is the margin of error, which is 5% (0.05).

Therefore,

$$\begin{array}{c}n=\frac{{\left(1.96\right)}^{2}0.5(1-0.5)}{0.0025}\\ n=384.16\end{array}$$

Therefore, the minimum sample size for this study was 384 patients approximated to be 422 after adjustment for a 10 percent non response rate.

### Quantitative sampling

The systematic sampling process was designed to select 412 patients for interviews for working 10 days, with a daily minimum patient arrival of 500 patients. The daily interview target was calculated by dividing the total number of patients (412) by the number of days (10), resulting in an average of 41.2 interviews per day.

The systematic sampling process began with setting up a consent desk and queue number system. Patients were informed about the survey, and consent was obtained. Each patient was assigned a unique queue number upon arrival.

To determine the sampling interval, the total daily patients (500) were divided by the daily interview targets (41 or 42 patients). This resulted in a sampling interval of approximately 12. A random starting point between 1 and 12 was selected, and from this point, every 12th patient was chosen for the interview.

For the daily interview allocation, 42 patients were interviewed on the first 5 days, and 41 patients were interviewed on the remaining 5 days. This method ensured an even distribution of interviews and a representative sample for the survey.

### Qualitative sample size

This study adopted a sample size of 12 respondents for the qualitative data collection, because it has been suggested that in practical research data saturation in a relatively homogeneous population can be achieved with this sample size [28]. Therefore, twelve (12) healthcare providers at the OPD and twelve (12) patients were selected making a total sample size of 24 for qualitative study.

### Qualitative sampling

To select 12 healthcare providers purposive sampling was employed. We targeted specific roles to ensure a comprehensive representation of the outpatient department: doctors, nurses, management, cashiers, and medical records personnel. The selection included 3 doctors, 3 nurses, 2 management personnel, 2 cashiers, and 2 medical records personnel. Doctors were chosen based on their direct patient interaction and diverse specializations within outpatient care. Nurses were selected to represent varying levels of experience, from junior to senior roles. Management personnel were chosen for their administrative and operational oversight responsibilities. Cashiers who handle patient transactions and medical records personnel involved in managing patient records were also included. This purposive sampling strategy aimed to capture a holistic view of the outpatient department's operations and challenges, providing valuable insights for the study. Also to select 12 patients we used convenience sampling. We chose individuals based on their accessibility and willingness to participate at the outpatient department. This approach involved approaching patients who were readily available and consented to participate in the study. The sampling process took place over several days, with researchers stationed in the waiting area to identify potential participants. Patients were approached in a systematic manner, ensuring a mix of different ages, genders, and medical conditions to achieve a varied sample. Each patient was briefly informed about the study's purpose and asked for their consent to participate. Those who agreed were included in the sample until the target of 12 patients was reached. This method was chosen for its practicality and ease of implementation, allowing researchers to quickly gather insights from a diverse group of patients without the need for complex selection criteria.

### Inclusion criteria

The study focused on patients aged 18 and older who attended the OPD during the data collection period.

### Exclusion criteria

Patients below 18 years or who were severely ill or had scheduled admission appointments were excluded, as well as first time attendees (new patients) because they lacked prior experience with the implemented interventions.

### Data collection tools and procedures

The researcher developed a structured questionnaire as a data collection tool. The tool had socio-demographic characteristics which included age, gender, marital status, education level, occupation, place of address, mode of payment and year of attendance at KCMC. The measurement scale for technical strategy was typically ordinal, based on fourteen (14) Likert scale questions with response options of 1 = strongly disagree, 2 = disagree, 3 = neutral, 4 = agree and 5 = strongly agree. Allowing patients to indicate their level of agreement or disagreement with statements related to technical strategies. Additionally, strongly disagree and disagree were consolidated as disagree and neutral, agree and strongly agree were consolidated as agree following the approach used in a previous study [29]. The internal reliability of the fourteen items used to assess effectiveness of technical strategies on reducing patient waiting time was measured using Cronbach’s alpha which was found to be 0.940. The survey included questions on arrival time, time the queue number was issued, registration waiting time, payment waiting time, triage waiting time, waiting time to see the doctor, pharmacy waiting time, laboratory waiting time, radiology waiting time and exit time. This data was collected from patients who attended clinics such as the general OPD clinic, orthopedic clinic, Medical clinic, surgical clinic, Urology clinic, Ear, Nose & Throat, Diabetic, cardiac clinic, Neurology and Neurosurgery. Waiting time was measured with a stopwatch.

Semi-structured guides for conducting in-depth interviews with patients and healthcare providers were developed. The interview guide had questions on socio-demographic and technical strategies such as the new block appointment system, use of EMR, extension of clinic days and availability of modern diagnostic equipment.

Also, the researcher conducted a documentary review, analyzing written records detailing time allocation before the studied event. This approach offered insights into past practices, aiding pattern and trend analysis. It involved reviewing benchmarks like a six-hour average waiting time, median waiting time for specific clinics, and total treatment duration for patients in various clinics. The six-hour benchmark was derived from the Ministry of Health's assessment report on OPD waiting time at KCMC and patients' information was not matched or linked to this report. Therefore, we considered the six-hour mark as our reference point." The data collection was conducted for two consecutive weeks from 3rd July to 14th July 2023.

### Data analysis

#### Quantitative data

The data collected were imported to the STATA programme (version 18.0) for further analysis. Descriptive Statistics: The analysis began with the presentation of data using various methods, including figures, graphs, and frequency distributions. The effect each response was rated on a scale of 1 to 5. Subsequently, cut-off points were utilized for each area to categorize the effectiveness of each intervention strategies as follows: 1–1.8 (very low), 1.8–2.6 (low), 2.6–3.4 (medium), 3.4–4.2 (high), and 4.2–5 (very high) [30]. Also, in this study, efficacy was determined by calculating the percentage reduction in OPD waiting time achieved through the implementation of intervention strategies. The current overall OPD waiting time (as shown in Table 4) was used as the numerator and the 6-h benchmark as the denominator [2].

The study defined the dependent variable as follows: overall patient waiting time, which was captured using a stopwatch, was categorized as a binary dummy variable. A value of 1 represented OPD waiting time less than 3 h, while a value of 0 indicated OPD waiting time exceeding 3 h. Comparison with Standards: The analysis involved evaluating OPD waiting time against established benchmarks. This included comparing the waiting time with the standards outlined in the Patients Charter of the United Kingdom (UK) and the recommendations from the United States Institute of Medicine (IOM), which advocate that at least 90% of patients should receive medical care within 30 min of their scheduled appointment time. Additionally, the study compared the observed 6-h waiting time, set as outpatient waiting time at KCMC Zonal Hospital, to assess whether there was any reduction post-intervention. Statistical Tests: To explore potential associations between dependent and independent variables, statistical tests were employed. Logistic regression analysis, encompassing both bivariate and multivariate analyses, was conducted. The multivariable analysis included all variables with *p* < 0.200 as identified during the bivariable analysis. It was further adjusted for sex, level of education, and mode of payment. All statistical analyses were conducted at a significance level of 0.05. These analytical steps were taken to provide a comprehensive assessment of the effect of the intervention on patient waiting time.

#### Qualitative data

All interview transcripts were transcribed verbatim and translated into English. In order to maintain the original meaning back translation was employed. The analysis was done using the English transcript. Thematic data analysis was employed using both deductive and inductive reasoning. Consequently, a preliminary codebook for data analysis was developed, aligning with the study objectives, after which the final codebook was imported into Atlas.ti 7.0 qualitative data analysis computer software. Inductive coding was assigned to text segments which built on emerged new themes that were not pre-determined. The codes were sorted into categories then were clustered into sub-themes which were aligned into themes. The entire process of analysis was iterative. In ensuring rigor, validity, and the mitigation of bias in the qualitative component, it was considered important to ensure the credibility, transferability, dependability, and confirmability of qualitative component to enhance its trustworthiness [31, 32]. In this study, credibility ensured that the data accurately reflects the real experiences and perceptions of those involved in the waiting process, allowing for subsequent decision-making. Transferability sought to make the findings relevant and to be applied to various healthcare settings beyond the specific study setting, ensuring that solutions can be adapted and implemented effectively in different contexts. Dependability ensured that the methods used to reduce waiting time were consistent and reliable over time, thus enabling the replication of the study's results. Confirmability ensures that the strategies for reducing waiting time are grounded in the data collected, rather than being influenced by the researchers' biases, thus enhancing the trustworthiness and effectiveness of the research findings in addressing waiting time issues in healthcare settings, thereby increasing the objectivity and validity of the research.

#### Ethical clearance

The Clearance Committee from Mzumbe University from the Directorates of Research, Publication and Postgraduate Studies provided ethical clearance with reference number MU/DPGS/INT/38/Vol. IV/236. Subsequently, the proposal was submitted for evaluation to the College Research Ethics and Review Committee (CRERC) at Kilimanjaro Christian Medical University College – Moshi. The CRERC granted approval, as indicated by certificate number 2639. Additionally, the data collection procedure received endorsement from the directors of KCMC Hospital reference number KCMC/P.1/Vol. XII. Prior to data collection, participants provided written informed consent. To ensure respondents’ autonomy, patients were fully informed about the purpose and nature of the study and provided with the option to withdraw at any time without any impact on their medical care. Patients were then questioned after completing their medical care. Also interviews were conducted in a private office within the OPD premises.

## Results

In this study, the initial calculated sample size was 422 patients. However, out of this group, only 412 patients consented to participate and completed the questionnaire. This resulted in a response rate of 97.6%. The median age was 52 (IQR, 38–65), with the majority aged over sixty. Over half were female (53.6%, *n* = 221), and the majority were married (76%, *n* = 313). Most had a basic education, including primary (44.7%, *n* = 184) and secondary education (26.7%, *n* = 110). More than half were peasant farmers (52.4%, *n* = 218), and the vast majority (94.7%, *n* = 338) resided within the KCMC catchment area. The majority were insurance patients (82.0%, *n* = 338), and more than two-thirds (66.5%, *n* = 274) had attended KCMC before the intervention's inception (Table 1).

**Table 1:** Socio-demographics characteristics of patients in the quantitative sample

Variable	n	Percentage (%)
**Gender**		
Male	191	46.4
Female	221	53.6
**Age-group**		
18 – 35	89	21.6
36 – 45	70	17.0
46 – 60	114	27.7
61 +	139	33.7
**Age (Median, [IQR])**	**52, [38—65]**	
**Marital status**		
Married	313	76.0
Single	64	15.5
Divorce/Separated	4	1.0
Widow/widower	31	7.5
**Level of education**		
Not attended school	20	4.9
Primary	184	44.7
Secondary	110	26.7
College	61	14.8
University	37	9.0
**Profession/Care**		
Peasant farmer	218	52.4
Business	80	19.4
Employed	86	20.9
Self-employed	20	4.9
Student	10	2.4
**Residence**		
Within KCMC Catchment area	390	94.7
Outside KCMC Catchment area	22	5.3
**Mode of payment**		
Cash	70	17.0
Insurance	338	82.0
Exempted	4	1.0
**First Year of attendance at KCMC**		
2018 and below	164	39.8
2019	34	8.3
2020	33	8.0
2021	43	10.4
2022	54	13.1
2023	84	20.4
**Patient attendance at KCMC**		
After intervention	138	33.5
Before intervention	274	66.5

### Demographic characteristics in the qualitative sample for healthcare providers

A total of 12 healthcare providers were enrolled of whom half were male (50%, *n* = 6) and half (50%, *n* = 6) were female (Table 2).

**Table 2:** Demographic characteristics in the qualitative sample for healthcare providers

Variable	n	Percentage (%)
**Gender**		
Male	6	50
Female	6	50
**Age-group**		
36 – 45	4	33.3
46 – 60	8	66.7
**Marital status**		
Married	14	100
**Level of education**		
College	5	41.7
University	7	58.3

### Demographic characteristics in the qualitative sample for patients

A total of 12 patients were enrolled of whom half were male (50%, *n* = 6) and half (50%, *n* = 6) were female (Table 3).

**Table 3:** Demographic characteristics in the qualitative sample for patients

Variable	n	Percentage (%)
**Gender**		
Male	6	50.0
Female	6	50.0
**Age-group**		
36 – 45	2	16.7
46 – 60	6	50.0
61 and above	4	33.3
**Marital status**		
Married	14	100
**Level of education**		
Primary	1	8.3
Secondary	1	8.3
College	6	50.0
University	4	33.3
**Occupation**		
Employed	10	83.3
Self-employed	2	16.7
**Residence**		
Within KCMC Catchment area	14	100

### Sub-themes from the in-depth interviews

During IDIs sub-themes that emerged were; ownership, training, organization culture, ineffective follow up, effective follow up and enhanced process simplification (Table 4).

**Table 4:** Themes that emerged from the IDIs

Themes	Sub-themes
Block appointment	∙ Ownership ∙ Training ∙ Organizational culture ∙ Ineffective follow up
Electronic medical records	∙ Effective follow up
Extension of clinic days	∙ Ownership ∙ Training ∙ Organizational culture ∙ ineffective follow up
Availability of modern diagnostic equipment	∙ Enhanced process simplification

### OPD waiting time since the inception of implementation of the interventions

Following the intervention, the overall median waiting time in the OPD was 3.30 h IQR (2.51–4.08) a reduction of 2.30 h after the intervention.

The median waiting time for registration was 9 min IOR (0.03–0.15). For payment, the median waiting time was 10 min IOR (0.07–0.15). For triage patients using out-of-pocket payments experienced median waiting time of 17 min IQR (0.05–0.19) while those with insurance had median waiting time of 14 min IQR (0.06–0.19) and the median waiting time to see a doctor was 1.36 h IQR (0.51–2.01). The time from arrival to actually seeing a doctor was measured at 3.08 h IQR (2.13–3.30). Furthermore, the median consultation time was 19 min IQR (0.15–0.24), waiting time at the pharmacy was 4 min IQR (0.02–0.06), at the laboratory it was 31 min IQR (0.20–0.37) and waiting time at Radiology varied based on the specific service. X-ray services in different rooms had average waiting time ranging from 35 min to 1.15 h with varying IQR (0.23–2.19). Ultrasound services had median waiting time of 32 min (Table 5).

**Table 5:** Distribution of Patient waiting time according to service areas

Variable	Hours Median [IQR]
Average waiting time for registration	0.09[0.03–0.15]
Average waiting time for payment	0.10[0.07–0.15]
Average waiting time to receive triage services	
Out of pocket	0.17[0.05–0.19]
Insurance	0.14[0.06–0.19]
Average waiting time to see the Doctor	1.36[0.51–2.01]
Average waiting time from arrival to see a doctor	3.08[2.13–3.30]
Average consultation time	0.19[0.15–0.24]
Average waiting time at the Pharmacy	0.04[0.02–0.06]
Average waiting time at Laboratory	0.31[0.20–0.37]
Average waiting time at Radiology	
X-ray (room 4)	0.35 [0.23–1.1]
X-ray (room 6)	1.22[0.39–2.10]
X-ray (special)	1.15[1.10–2.19]
Ultrasound	0.32[0.26–0.41]
Overall waiting time (arrival to the exit)	3.30[2.51–4.08]

### Qualitative findings

#### Registration (medical records department)

The adoption of electronic medical records (EMRs) appears to have enhanced the overall efficiency of the KCMC OPD registration process, benefiting both patients and staff.

*"I have been receiving treatment here at KCMC for over 20 years. In the past, in the medical records department, it was necessary to have someone, a staff member, whom you would contact in advance, preferably three days before your clinic day, so that they could start looking for your file. This way, you could save time waiting. However, nowadays, this process is no longer in place. When I arrive, I simply present my card, and in no time, I'm on my way to the next area. There's no longer any time wasted at the reception."* (IDI – Male Patient, aged 67 years)

Another interviewee added that:

*"Nowadays, with the system in place, the process is streamlined, allowing me to efficiently register as many patients as possible in a short amount of time. I no longer have to leave the reception area to search for files, which has significantly improved the efficiency of the registration process."* (IDI – Male healthcare provider (HCP), aged 45 years)

#### Waiting time to see the doctor

The issue of waiting time for patients to see the doctor has emerged as a significant concern within the healthcare facility. This concern is consistently echoed in both the quantitative data and qualitative interviews.

For example a female HCP aged 40 years reported:

*"*[…] *commencing clinics promptly can be challenging for doctors, as it is crucial for them to first participate in the morning report, which provides essential updates on the status of hospitalized patients."* (IDI – male HCP, aged 40 years).

After probing as to why the medical staff cannot split into two teams of doctors so that one team could attend to outpatients the response was as follows:

*"We have a limited number of doctors, making it challenging to divide them into two groups. Moreover, admitted patients demand our additional attention, as some rely on oxygen for breathing, while others are too ill to walk. Unlike outpatients, the majority of whom can independently come for treatment, we kindly request their understanding as we prioritize the care of our admitted patients."* (IDI – male HCP, aged 40 years).

A female patient aged 53 years gave some observations.

*“[….] Mmh! I want to highlight that delay in seeing the doctor can have serious consequences. It can lead to a worsening of symptoms or conditions, increase stress levels, and ultimately result in reduced satisfaction with the healthcare service. It's imperative that we address these extended waiting times. This is crucial not just for the comfort of the patient, but also to ensure that medical care is administered in a timely and effective manner.”* (IDI – female patient, aged 53 years).

#### Pharmacy

In the pharmacy department, there has been a notable improvement in waiting time. Patients now experience a comfortable and efficient process, with minimal time spent before receiving their prescribed medications.

*"With the use of a computerized system, things have been greatly simplified. The waiting time to collect medicine has become short. When I come here, I wait for just a little while and quickly get my medicine."* (IDI – Male patient, aged 45 years).

*“Apart from using the computerized system in place, which has simplified things, the hospital administration has managed to establish three additional pharmacies apart from this one, thus reducing congestion in a single pharmacy, as it used to be in the past. That's why now a patient can be served quickly.”* (IDI – male HCP, aged 50).

#### Laboratory department

In the laboratory department, the waiting time has been a subject of varying experiences among patients. Some patients have reported relatively short waiting periods, while others have encountered longer waits.

*“I have been patiently waiting for a long time to be called for my tests, I’ve not yet been called up to now.”* (IDI – female patient, aged 43 years).

Another interviewee shared that:

*"I've noticed that one of the main reasons for long waiting time at the laboratory here is the limited space. The laboratory rooms at the Outpatient Department (OPD) have remained the same since the hospital was established, which means they can only accommodate a small number of patients at a time. This often leads to a backlog of patients waiting to get their tests done. It's clear that expanding the laboratory facilities is crucial to reduce these extended waiting time and ensure more efficient service delivery for everyone”* (IDI – male HCP, aged 55 years).

#### Radiology department

Despite having modern diagnostic equipment, which appears to have significantly contributed to reducing patient waiting time, there are still instances where patients experience long waiting time in the radiology department.

*"For me, even though waiting for an X-ray may take some time, I don't mind the wait. I've noticed a significant improvement in waiting time compared to before. In addition nowadays, when I have an X-ray, I can also consult with my doctor on the same day, which wasn't possible in the past”* (IDI – male patient, aged 40 years).

One interviewee highlighted a crucial factor contributing to the extended waiting time at the radiology department and pointed out that:

*“The same rooms at the radiology department are utilized for both outpatient and inpatient cases. As a result, priority is often given to the admitted patients, leading to longer waiting time for those seeking outpatient radiology services. This dual-use of facilities poses a challenge in managing patient flow and significantly contributes to the observed delays in the radiology department”.* (IDI – female HCP, aged 49 years)

#### Patient OPD waiting time with Six (6) and Three (3) Hours Threshold

**Fig. 1:**
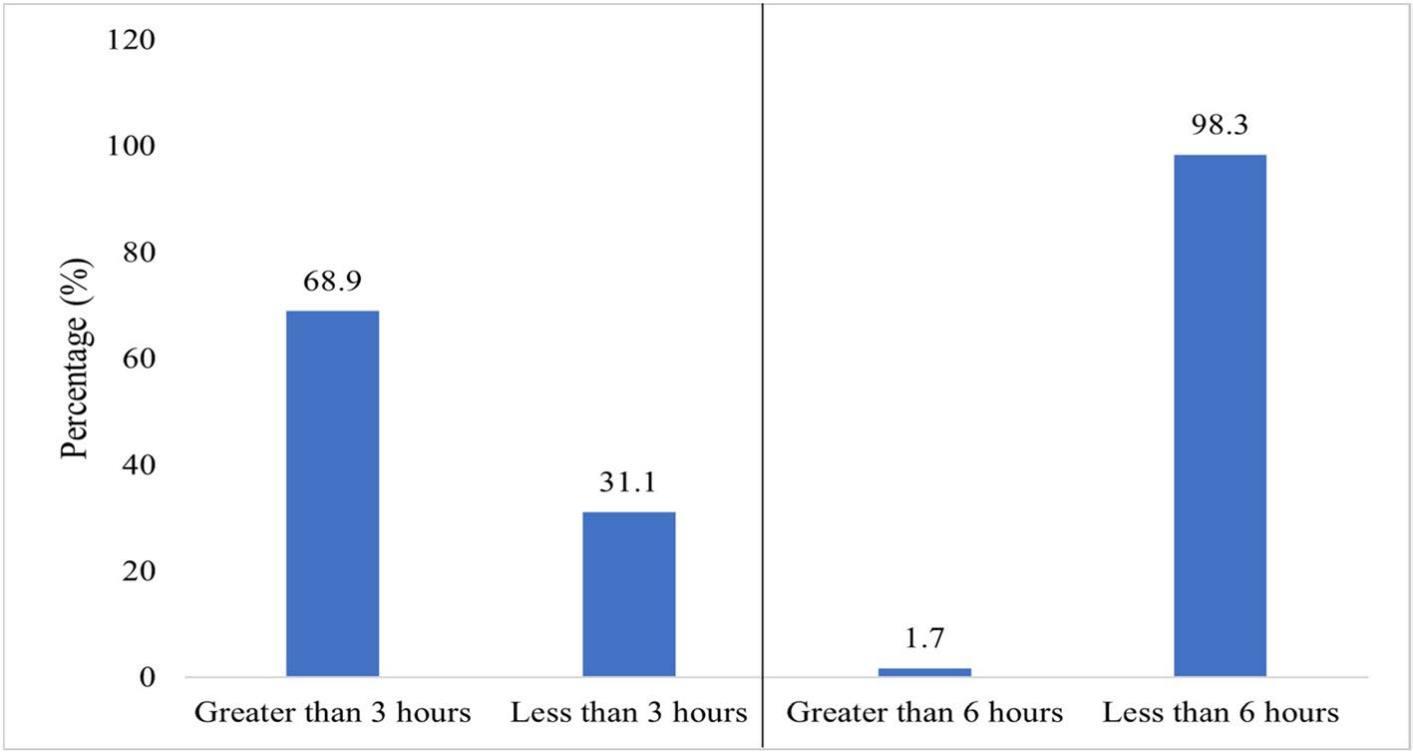
Patient OPD waiting time with six (6) and three (3) hours threshold (*n* = 412)

Not a single patient managed to complete the treatment within the recommended 30-min window following their scheduled appointment. When assessed based on the KCMC benchmark of a 6-h timeframe, the vast majority of patients (98.3%, *n* = 407, 95% CI, 97.0%-99.5%) indicated that they received the OPD services within a period of less than six hours. However, when the time threshold was further reduced to three hours, 31% (*n* = 128, 95% CI, 26.6%-35.6%) of all surveyed patients reported that they received OPD services within a duration of fewer than three hours (Fig. 1).

### Qualitative findings

Furthermore, during the in-depth interviews (IDIs), patients emphasized receiving OPD services within a timeframe of below three hours.

For instance, a 58-year-old female patient remarked:

“*Certainly, drawing from my extensive experience of over 15 years attending KCMC hospital, I can attest to the positive changes in the waiting time for OPD services. Patients, including myself, are genuinely appreciative of this effective reduction in waiting time. I personally find it remarkable that I can now complete all the necessary OPD services in just about three hours, which is a stark contrast to the longer waiting periods we used to endure. This improvement has undoubtedly enhanced the overall patient experience and contributes positively to our healthcare journey”.* (IDI – female patient, aged 58 years)

### Effect of technical strategies on patient waiting time

#### Descriptive statistics of the technical strategies

The study assessed the effectiveness of various technical strategies on reducing patient waiting time, categorized into four domains: block appointment, implementation of electronic medical records (EMR), extension of clinic days throughout the week, and utilization of modern diagnostic tools. The self-reported data were analyzed using mean scores and standard deviations, measured on a Likert scale ranging from 1 (strongly disagree) to 5 (strongly agree). The effectiveness of strategies in reducing patient waiting time was categorized as follows: very low (1–1.8), low (1.8–2.6), medium (2.6–3.4), high (3.4–4.2), and very high (4.2–5).

Overall, the average effectiveness of technical strategies in reducing patient waiting time was found to be very high with a mean score of 4.27 (SD = 0.904) with a descriptive equivalent of “very high”. Specifically, the new block appointment system obtained a mean score of 4.36 (SD = 0.856) with a descriptive equivalent of “high”. Additionally, the introduction of hourly appointments demonstrated positive effects with a mean score of 4.18 (SD = 1.024) with a descriptive equivalent of “high”. The transition from paper based to electronic medical records was also effective and obtained a mean score of 4.09 (SD = 1.033) with a descriptive equivalent of “high”. Moreover, the extension of clinic days obtained a mean score of 4.31 (SD = 0.832) with a descriptive equivalent of “very high”. Finally, the availability of modern diagnostic services, achieving a mean score of 4.30 (SD = 0.861) with a descriptive equivalent of “very high” (Table 6).

**Table 6:** Technical Strategies towards Patient Waiting Time

Statement	Level of agreement					Mean (± SD)
	**SD** **n (%)**	**D** **n (%)**	**N** **n (%)**	**A** **n (%)**	**SA** **n (%)**	
**Block appointment**						
New Block appointment system	7(1.7)	14(3.4)	19(4.6)	156(37.9)	216(52.4)	4.36(0.856)
I prefer the block appointment system to single appointment	12(2.9)	20(4.9)	19(4.6)	180(43.7)	181(43.9)	4.21(0.949)
Hourly appointment reduces waiting time	11(2.7)	23(5.6)	22(5.3)	170(41.3)	186(45.2)	4.21(0.963)
Block appointment distributes patient arrival more evenly	19(4.6)	18(4.4)	17(4.1)	174(42.2)	184(44.7)	4.18(1.024)
**Overall Mean**						**4.24(0.948)**
**Electronic Medical Records**						
Drawbacks of using paper based are outweighed by EMR	8(1.9)	12(2.9)	21(5.1)	167(40.5)	204(49.5)	4.33(0.855)
EMR improves communication between healthcare providers	6(1.5)	17(4.1)	17(4.1)	173(42.0)	199(48.3)	4.32(0.850)
Easy access to patient information with EMR	8(1.9)	16(3.9)	18(4.4)	181(43.9)	189(45.9)	4.28(0.870)
Using EMR the time required to wait for documentation is decreased	10(2.4)	17(4.1)	17(4.1)	157(38.1)	211(51.2)	4.32(0.916)
Migrating from paper based to EMR reduces waiting time	20(4.9)	19(4.6)	26(6.3)	187(45.4)	160(38.8)	4.09(1.033)
**Overall Mean**						**4.27(0.905)**
**Extension of Clinic days**						
Extension of clinic days reduces waiting time	7(1.7)	11(2.7)	23(5.6)	177(43.0)	194(47.1)	4.31(0.832)
Extension of clinic days gives one additional options	8(1.9)	15(3.6)	19(4.6)	174(42.2)	196(47.6)	4.30(0.869)
**Overall Mean**						**4.31(0.851)**
**Modern Diagnostic services/equipment**						
Availability of modern diagnostic services has reduced waiting time	7(1.7)	13(3.2)	28(6.8)	164(39.8)	200(48.5)	4.30(0.861)
Digitalizing diagnostic machines reduces waiting time	12(2.9)	11(2.7)	22(5.3)	168(40.8)	199(48.3)	4.29(0.908)
Computerization has helped to reduce registration time	14(3.4)	14(3.4)	25(6.1)	147(35.7)	212(51.5)	4.23(0.969)
**Overall Mean**						**4.27(0.913)**
**Grand overall mean**						**4.27(0.904)**

*SD* Strongly disagree, *D* disagree, *N* Neutral, *A* Agree, *SA* Strongly agree

### Bivariable analysis of technical strategies and patient waiting time

Bivariable regression analysis established a significant association between new block appointment system (OR 3.34; CI 1.28–8.77: *p* = 0.014), hourly appointment system (OR 2.49; CI 1.01–6.13; *p* = 0.047) and patient waiting time (Table 7).

**Table 7:** Bivariable analysis of technical strategies and patient waiting time

Variable	Patient Waiting Time		OR, 95% CI	*p*-value
	More than 3 h N (%)	Less than 3 h N (%)		
**New Block appointment system**				
Disagree	34(87.2)	5(12.8)	1	
Agree	250(67.0)	123(33.0)	3.34, 1.28–8.77	**0.014**
**I prefer the block appointment system to single appointment**				
Disagree	25(78.1)	7(21.9)	1	
Agree	259(68.2)	121(31.8)	1.67, 0.70–3.96	0.246
**Hourly appointment reduces waiting time**				
Disagree	31(83.8)	6(16.2)	1	
Agree	253(67.5)	122(32.5)	2.49, 1.01–6.13	**0.047**
**Block appointment distributes patient arrival more evenly**				
Disagree	26(76.5)	8(23.5)	1	
Agree	258(68.3)	120(31.8)	1.51, 0.66–3.43	0.324
**Drawbacks of using paper based are outweighed by EMR**				
Disagree	17(80.9)	4(19.1)	1	
Agree	267(68.3)	124(31.7)	1.97, 0.65–5.99	0.230
**EMR improves communication between healthcare providers**				
Disagree	15(65.2)	8(34.8)	1	
Agree	269(69.2)	120(30.9)	0.84, 0.35–2.03	0.692
**Easy access to patient information with EMR**				
Disagree	14(70.0)	6(30.0)	1	
Agree	270(68.9)	122(31.1)	1.05, 0.39–2.81	0.916
**Using EMR the time required to wait for documentation is decreased,**				
Disagree	16(66.7)	8(33.3)	1	
Agree	268(69.1)	120(30.9)	0.89, 0.37–2.15	0.805
**Migrating from paper based to EMR reduces waiting time**				
Disagree	14(51.9)	13(48.2)	1	
Agree	270(70.1)	115(29.9)	0.46, 0.21–1.01	0.052
**Extension of clinic days reduces waiting time**				
Disagree	11(61.1)	7(38.9)	1	
Agree	273(69.3)	121(30.7)	0.69, 0.26–1.84	0.466
**Extension of clinic days gives one additional options**				
Disagree	15(65.2)	8(34.8)	1	
Agree	269(69.2)	120(30.9)	0.84, 0.35–2.03	0.692
**Availability of modern diagnostic services**				
Disagree	12(60.0)	8(40.0)	1	
Agree	272(69.4)	120(30.6)	0.66, 0.26–1.66	0.379
**Digitalizing of diagnostic machines reduces waiting time**				
Disagree	16(69.6)	7(30.4)	1	
Agree	268(68.9)	121(31.1)	1.03, 0.41–2.57	0.946
**Computerization has helped to reduce registration time**				
Disagree	19(67.9)	9(32.1)	1	
Agree	265(69.0)	119(31.0)	0.95, 0.41–2.16	0.899

#### Multivariable analysis between technical strategies and patient waiting time

Multivariable logistic analysis was employed to determine which technical strategy played a significant role in reducing patient waiting time. The findings in the adjusted odds ratio indicate that there was an association between reduction of patient waiting time and migrating from paper based to electronic medical records, thus electronic medical records remained a significant factor for patient waiting (AOR = 2.08, 95% CI, 1.10–3.94, *p*-value = 0.025). However, the introduction of the new block appointment system demonstrated a higher likelihood for a positive effect on reducing waiting time, although the findings were not statistically significant (AOR = 2.49; 95% CI, 0.68–9.10, *p*-value = 0.168) (Table 8).

**Table 8:** Multivariable analysis of technical strategies and patient waiting time

Variable	AOR, 95% CI	*p*-value
**Sex**		
Male	1	
Female	0.79, 0.51–1.23	0.296
**Education**		
Non formal education	1	
Basic education	2.60, 0.71–9.45	0.147
Collage and above	2.98, 0.78–11.37	0.110
**Mode of payment**		
Cash	1	
Insurance	1.64, 0.61–4.38	0.324
**New Block appointment system**		
Disagree	1	
Agree	2.89, 0.83–10.13	0.097
**Introduction of hourly appointment**		
Disagree	1	
Agree	2.49, 0.68–9.10	0.168
**Migrating from paper based to EMR**		
Disagree	1	
Agree	2.08, 1.10–3.94	**0.025**

### Qualitative findings with regards to technical strategies

#### Block appointment

Based on the findings from in-depth interviews, both patients and healthcare providers expressed varying opinions on the block appointment system.

One female patient aged 62 years said that:

*"Over the years, I have become accustomed to coming in the morning. I can't come at other time besides the morning; it would disrupt my plans."* (IDI, female patient, aged 62 years).

A male healthcare provider aged 39 years shared the experience:

“*The truth is, we haven't been very successful in using block appointments. We tried it on the first and second days, but things went back to how they were before. The problem is, patients arrive very early in the morning, and you find them all crowded, waiting for service. Once a patient arrives, they must be attended to. We've realized that this block appointment system requires the whole team to be involved, from medical records (reception) to doctors, nurses, and the patients themselves”* (IDI – male HCP, aged 51 years).

However, it's important to note that amidst these negative perspectives, several interviewees also acknowledged the positive effect of the system.

“*Since the introduction of the appointment system in 2020, we've observed a significant reduction in patient waiting time, which has led to quicker and more efficient service delivery for patients. When a patient arrives, the waiting area is usually less crowded. Furthermore, doctors now have more spaced-out appointments, allowing them to devote ample time to each patient.”* (IDI – female HCP, aged 47 years).

#### Electronic medical records

The quantitative finding regarding migrating from paper based to electronic medical records aligns with our qualitative findings.

*"From my experience, dealing with physical files presented its own set of challenges. There was a lengthy process, and files were prone to being misplaced, including important test results. Sometimes, files would be delayed in reaching the clinic. This was particularly problematic for patients who arrived early; if their files couldn't be located promptly, it would cause a delay. However, with the new system in place, everything operates swiftly and efficiently. The system has truly revolutionized the process”* (IDI – female HCP, aged 55 years)

One interviewee shared the experience.

*“When I come for treatment nowadays, I no longer experience the frustration of my test results going missing or my file being unavailable."* (IDI – male patient, aged 50 years)

#### Extension of clinic days

The implementation of the daily clinic schedule has yielded mixed results.

One interviewee stated that:

*"In our department, the limited number of staff has posed a challenge. Conducting daily clinics becomes demanding, as the same doctor is tasked with conducting ward rounds, making decisions for admitted patients, and performing surgery. However, once we have an adequate staff complement, we can begin seeing patients on a daily basis."* (IDI – male HCP, aged 42 years)

On the contrary, extension of clinic days has proven to be a highly beneficial strategy in our facility serving as one of the key strategies to address patient waiting time.

*“It has significantly reduced the patient waiting time. In the past, clinics used to run until 6 pm in the evening. Since they implemented the daily clinic schedule, patients are now seen earlier, and the clinics end earlier. This is because patients have been scheduled throughout the week”.* (IDI – female HCP, aged 48 years)

Another interviewee supported that.

*“It has helped in limiting the number of patients flocking to a single clinic, but it doesn't necessarily reduce patient waiting time."* (IDI – male HCP, aged 51 years)

One interviewee shared the experience:

*"These days, I finish my treatments earlier than I used to.”* (IDI – male patient, aged 49 years)

#### Availability of modern diagnostic equipment

The integration of modern diagnostic equipment stands as a substantial contributor to the reduction of patient waiting time. This positive trend is supported by both our quantitative and qualitative findings, affirming the significance of having advanced diagnostic tools readily accessible within our healthcare facility.

"*Nowadays, the procedure has become significantly more simplified. You just need to consult the system to retrieve the patient's results. When you open it, you can readily peruse the information, making the process more efficient. If I require additional specifics about the condition, it's easy to locate them in the patient's file. I simply access it in the system, and their image is readily available, leading to a substantial time-saving."* (IDI – male HCP, aged 40 years)

More experience is shared from a male patient aged 45 years.

*"I now do my investigations on the same day and return to the doctor for my results. This contrasts with the past when I needed to be scheduled for a different day to pick up the results. This has resulted in a considerable time-saving."* (IDI – male patient, aged 45 years)

## Discussion

### OPD waiting time

Following the intervention, it was observed that the overall median waiting time in the OPD was reduced to 3.30 h in contrast to the previous six-hour (6) waiting time prior to the intervention, showing the effectiveness of the intervention achieving a reduction of waiting time by 45%. This improvement is significant and suggests that the interventions have had a positive effect.

These findings align with other research involved adding more human resources and changing business and management practices. The findings demonstrated a significant success in reducing wait time in the USA, China, Sri Lanka and Taiwan by 15%, 78%, 60%, and 50%, respectively [33].

The study at KCMC found low median waiting time of 9 min for registration. This is not congruent with findings in China and Saudi Arabia where registration time were notably higher [24, 34]. In Ethiopia, waiting time varied, with some patients waiting over an hour [35], while another study reported a median wait of 18 min [36]. In Kenya, registration waiting time were even shorter 5.8 min [37]. These discrepancies could be explained by variations in patient flow management techniques or data collection techniques. Overall, the study shows that the waiting time for registration has significantly decreased at KCMC, clearly demonstrating the efficiency of the technical strategies that have been put in place to cut down on waiting time.

In terms of payment processing, the median waiting time was 10 min. Although it appears majority of patients were insured, the mode of payments had no significant association with waiting time. This suggests that insured patients were handled just as quickly as patients paying with cash. These results are relatively congruent with a study conducted in a Tertiary Care Hospital in Pune, India, where patients spent an average of 7 min at the cashier [26]. This shared emphasis on streamlined payment processes underscores their significance in enhancing the patient experience, reinforcing the importance of efficient payment processing in healthcare settings.

At the triage area, patients paying cash had a median waiting time of 17 min, while insured patients experienced slightly shorter median waiting time of 14 min. These results are congruent with a study by [38], who found that insured patients at a hospital in Northeast Thailand had an average triage waiting time of 13 min. The consistency in findings between these studies suggests that insurance status may play a role in patient waiting time, with insured patients benefiting from somewhat more efficient service and well streamlined patient flow. However, it's important to note that regional contexts may influence waiting time, and these results may vary in different healthcare settings and countries.

The median waiting time before seeing a doctor from arrival to consultation was 3.08 h. These results resonate with research from Nigeria, where 38% of respondents waited for over 2 h for a consultation [39, 40] found an average waiting time of 137.02 ± 53.64 min before seeing a doctor. In contrast, some studies reported shorter waiting time, such as 40 min in India [26], over 90% of patients waiting for more than 20 min in Saudi Arabia [24] and more than half of patients waiting for over 60 min in Ethiopia [35]. Involvement of doctors in teaching students, long ward rounds, staff constraints and prioritizing inpatients over outpatients could all contribute to doctors coming late to the clinics, thus, causing increased stress, discomfort, and impatience among patients.

The study's findings emphasize a positive aspect of healthcare delivery at KCMC, specifically in pharmacy services, with remarkably short median waiting time of 4 min. This aligns with research in Iran by [41], which also reported the pharmacy as having the shortest average waiting time of 5 ± 3 min and in Kenya, where patients experienced a similar pattern with an average waiting time of 5.5 min [37]. However, these results contrast with a study in a Tertiary Care Hospital in Pune, India, revealing a 15-min average waiting time at the pharmacy [26]. In Ethiopia, [35] found that only 23.6% of patients received their prescribed drugs within ≤ 30 min, while a comparable number received them within 30–60 min or > 60 min. During interview, patients commended the computerized system's effectiveness in streamlining the medication collection process, which the study attributes to its implementation. In addition, three new pharmacies have been added to the existing one, reducing congestion and allowing patients to receive faster service. The aforementioned positive results serve as evidence of the efficiency with which KCMC's pharmacy services have integrated technology.

The median waiting time at the laboratory department was 31 min. This is congruent with studies done in Ethiopia which reported a similar median of waiting time of 31 min, reflecting consistency in laboratory waiting time within Ethiopian healthcare settings [36]. Similarly, another study noted that 58.1% of patients received laboratory services within 30 to 60 min, with only 12.0% within ≤ 30 min [35]. On the contrary, in Nigeria a study revealed a longer waiting time, with patients waiting over 50 min on average for laboratory services. This suggests that KCMC’s laboratory waiting time maybe more favourable when compared to other hospitals [42]. Nevertheless, another study reported an average waiting time which was significantly shorter, 12.75 min which suggest that there may be variations in waiting time between KCMC and Indian healthcare facility. The reason for the long waiting time at KCMC could be due to the limited space within the laboratory rooms resulting in the accommodation of fewer patients at any given time. This emphasized the necessity of expanding facilities to improve the effectiveness of service delivery [26].

Waiting time at the Radiology department showed significant differences depending on which investigation was ordered. Thus the median waiting time for X-ray services varied between rooms, from 35 min to 1.15 h, whereas the median waiting time for ultrasound services was 32 min. Important insights into patient experiences were obtained through in-depth interviews. Some patients expressed contentment with the waiting time for X-rays because they were able to get the results on the same day and continue with further treatment from their doctors on the very same day. Various studies revealed differing median radiology waiting time. Iran reported 27 min ± 11 [41] while India recorded 36.05 min [26], Ethiopia’s studies indicated 33 min [36] and 60 min [35], all indicating relatively shorter waiting time. Conversely, Nigeria showed the longest waiting time for radiological services at 77 min [42]. There were issues identified within KCMC's Radiology department, such as the dual use of rooms for outpatient and inpatient cases, which prioritized admitted patients and resulted in longer wait time for outpatients. This organizational practice complicates patient flow management and contributes considerably to perceived delays in the radiology department. The findings emphasize that waiting time in Radiology are influenced by resource availability, facility organization, and patient flow management.

### Technical strategies on patient waiting time

The implemented block appointment system appears to have the potential to improve waiting time, even though the effect was not statistically significant. Early patient arrivals continue to be problematic, which emphasizes how crucial it is to provide efficient patient education and coordination in order to reap the full rewards of this system. Similar findings in Nigeria demonstrate that appointments with specific time are uncommon, resulting in early patient arrivals and possible delays in the start of services [8]. However, in other nations where it has been used, the block appointment system has proved to be successful. Research conducted in the United States [43] and the United Kingdom [44] have demonstrated its effectiveness in reducing patient wait time. In Thailand [5] and Sri Lanka [7] demonstrated the possible advantages of carefully planned scheduling by demonstrating how the use of appointment systems can dramatically reduce average waiting time. Block appointment scheduling also successfully spread out patient arrivals throughout the day, as shown by a pilot study conducted in Mozambique, which significantly decreased waiting time [9]. Hence, coordinated efforts involving medical records, physicians, nurses, and patients themselves are needed to operate the system.

The transition from paper to electronic medical records had a significant and positive impact on reducing long waiting time at the OPD. Various studies underlined the benefits of electronic medical records over paper-based systems, including how it can improve patient waiting time, increase efficiency, and improve the delivery of healthcare services [10, 11] and [12]. Another study highlighted the preference for electronic health records among healthcare providers due to their efficiency and speed in patient care. By eliminating labour intensive procedures, space limitations, and document misplacement problems associated with manual filing systems, the switch to electronic records helped to create more efficient and productive operations [13]. The entire patient experience was greatly enhanced since patients were no longer frustrated by lost records or delayed test results. The implementation of electronic health records has proven to be beneficial in reducing extended wait time in outpatient clinics, as evidenced by a study carried out in Brazil [14].

The extension of clinic days yielded a mean score of 4.31 (SD = 0.832) signifying positive effect. Similarly, qualitative findings from healthcare providers and patients shed light on the effect of extending clinic days. The department's small staffing posed a significant challenge, as doctors had to manage multiple responsibilities, such as ward rounds, decision-making for admitted patients, and surgery. These findings are not congruent with those of other locations where clinic days have been extended. For instance, a study suggested that extending clinic days was more effective, resulting in a 26% reduction in average waiting time [15]. Additionally, another study found a significant 56% reduction in average waiting time after extending clinic days, coupled with high patient satisfaction rates [16]. Similarly in other study extending clinic days resulted in an astounding 46% decrease in average waiting time. The study also found that patient satisfaction was high and that the number of patients seen each day had increased [17].

The availability of modern diagnostic services had a mean score of 4.30 (SD = 0.861), signifying a positive effect. This demonstrates that advanced diagnostic equipment played a significant role in streamlining healthcare processes and enhancing efficiency. Qualitative findings from both healthcare providers and patients supported this, highlighting how digital systems and modern equipment simplified procedures and expedited healthcare services. Access to electronic patient information and test results contributed to time savings. These findings are congruent with studies conducted in Italy [18], Pakistan [19, 20], and Iran [21], which all demonstrated reductions in waiting time following the acquisition of modern equipment. A study from India also supported the positive impact of modern equipment on patient waiting time [22]. Additionally, audit assessments in Tanzania by the Ministry of Health and equipment-related observations in zonal hospitals emphasized the critical role of modern equipment in healthcare settings. Outdated equipment can lead to extended patient waiting time, underscoring the importance of maintaining and upgrading diagnostic facilities to improve healthcare efficiency and patient care [2].

## Conclusion

The implemented technical strategies resulted in a significant reduction in overall OPD waiting time to an average of 3.30 h, marking a 45% reduction from the previous six-hour wait. While there have been notable improvements in registration, payment, triage, and pharmacy services, issues remain in doctor consultations, laboratory, and radiology services, resulting in extended waiting time for some patients. The adoption of electronic medical records emerged as the most effective technical strategy, emphasizing its critical role in improving OPD efficiency. Despite these advancements, additional improvements are required to meet the global standard of waiting time ranging from 30 min to 2 h. Nevertheless, ineffective implementation of block appointment and extension of clinic days appears to stem from lack of ownership and proactive involvement by hospital managers in driving these strategies forward. Furthermore, the hospital's dominant organizational culture seemed to be resistant to change, which could hinder the effective implementation of these strategies. The results indicated a possible training shortfall, suggesting that personnel may not have had enough training to properly adopt and implement these new strategies. Moreover, there was a lack of effective follow-up and management strategies by hospital managers, potentially hindering the sustained implementation of these strategies. Moreover, the shared use of central modern diagnostic equipment between inpatient and outpatient services at the radiology department resulted in delays, impacting waiting time. Alongside, a comprehensive review of the diagnostic service structure might be necessary to alleviate delays and streamline services for both inpatient and outpatient care.

### Limitations of the study

Since only one hospital was involved in the study, generalization to cover the rest of Tanzania remains uncertain. Additionally, there was a chance that selection bias might have impacted the findings.

### Supplementary Information

Supplementary Material 1.

## Data Availability

Data is available upon request from the corresponding author.

## Abbreviations

CRERC: College research ethics and review committee
EMR: Electronic medical records
HCP: Healthcare provider
IDI: In-depth interview
IOM: Institute of medicine
IQR: Interquartile range
KCMC: Kilimanjaro Christian Medical Centre
MoHCDGEC: Ministry of health, community development, gender, elderly and children
MU: Mzumbe University
OPD: Outpatient department
