# Supplementary material for: Outcome evaluation of technical strategies on reduction of patient waiting time in the outpatient department at Kilimanjaro Christian Medical Centre—Northern Tanzania
Source: BMC Health Serv Res. 2024 Jul 9;24:785. doi: 10.1186/s12913-024-11231-5 (PMC11234691; doi:10.1186/s12913-024-11231-5)
Supplement: Supplementary file 1 — Supplementary Material 1. [file 12913_2024_11231_MOESM1_ESM.docx]

**QUESTIONNAIRE FOR PATIENTS**

My name is **Manasseh Joel Mwanswila** a student of MSc in Health Monitoring and Evaluation at Mzumbe University. I would like to ask you few questions about the intervention strategies implemented by the hospital to reduce the waiting time for outpatient healthcare services at KCMC Hospital.

**Title of the study:** I am conducting a study on ***“OUTCOME EVALUATION OF TECHNICAL STRATEGIES ON REDUCTION OF PATIENT WAITING TIME IN THE OUTPATIENT DEPARTMENT OF KILIMANJARO CHRISTIAN MEDICAL CENTRE - NORTHERN TANZANIA”*.** You have been selected as a participant in this study to give your views, opinions and experience and your participation is important as it will assist in improving care of patients. The results of his study will be used to provide information as a base especially in the study area, for researchers, to address the problem.

**Confidentiality:** All information obtained from you during the study will be kept strictly confidential, only the principal investigator will have access to it and will be used for research only and not for any other purpose.. Your name will not be mentioned in the study or in the report findings. Please feel free to participate.

**Voluntary consent**: Your participation in this research is entirely voluntary. Please feel free to ask any questions before or after the interview and you can choose not to answer some of the question, which you do not feel comfortable to answer. Additionally, you are free to decline to participate in the study at any time and you will not face any consequences if you do so.

Are you willing to participate YES NO

If YES: Signature……………………………………… Date ………………………………….

Researcher’s Name…………………………………………………………………………………

Signature……………………………………………… Date ……………………………………

**Thank you for your cooperation**

**QUESTIONNAIRE FOR PATIENTS (ENGLISH VERSION)**

**Hospital Registration Number……………………………… Date …………………………………..**

**Name of Clinic……………………………………………….. Day of the week…………………………..**

**Instruction:** Please tick the most appropriate answer (√)

**SECTION A: DEMOGRAPHIC INFORMATION**

1. Gender (tick in the box)

1. Male ( )
2. Female ( )

2. What is your age? ………………………….

3. What is your Marital Status?

1. Married ( )
2. Single ( )
3. Divorced/Separated ( )
4. Widow/Widower ( )

4. What is your level of education? Please tick in the box.

1. Not attended school ( )
2. Primary ( )
3. Secondary ( )
4. College (certificate or Diploma) ( )
5. University (Degree/Masters/PhD) ( )

5. What is your cadre/profession?

1. Peasant famer ( )
2. Business ( )
3. Employed ( )
4. Self-employed ( )
5. Student ( )

6. Residence

1. Within catchment area [Kilim anjaro, Arusha, Manyara, Tanga, Singida] ( )
2. Outside catchment area ( )

7. Mode of payment

- 1. Cash ( )
  2. Insurance ( )
  3. Exempted ( )

8. Since when did you start attending KCMC clinic

1. 2018 and below ( )
2. 2019 ( )
3. 2020 ( )
4. 2021 ( )
5. 2022 ( )
6. 2023 ( )

**Section B:** Please indicate your agreement level on the following statements by **ticking** (√) one of the options. **KEY: 1 = STRONGLY DISAGREE; 2 = DISAGREE; 3 = NEUTRAL (neither agree nor disagree); 4 = AGREE; 5 = STRONGLY AGREE**

| **Characteristics** | |  |  |  |  |  |
| --- | --- | --- | --- | --- | --- | --- |
| **Technical factor** | | **1** | **2** | **3** | **4** | **5** |
| 201 | The new Block appointment system has reduced waiting time |  |  |  |  |  |
| 202 | I prefer the block appointment system to the previous appointment system in terms of wait times. |  |  |  |  |  |
| 203 | Introduction of hourly appointment has helped to reduce waiting time |  |  |  |  |  |
| 204 | Distributing patient arrivals more evenly throughout the day help to reduce waiting times |  |  |  |  |  |
| 205 | Drawbacks of using paper based are outweighed by electronic medical records resulting in shorter waiting time |  |  |  |  |  |
| 206 | The electronic medical records system has improved the communication between healthcare providers, resulting in shorter waiting times |  |  |  |  |  |
| 207 | Easy to access patient information with electronic medical records system resulting in shorter waiting times |  |  |  |  |  |
| 208 | Using EMR the time required to wait for documentation is decreased, hence patient waiting time is reduced |  |  |  |  |  |
| 209 | Migrating from manual to electronic health record has proved to minimize prolonged waiting time at the OPD |  |  |  |  |  |
| 210 | Extension of clinic days has reduced waiting time |  |  |  |  |  |
| 211 | Extension of clinic days has given me additional options for clinic days to accommodate my schedule |  |  |  |  |  |
| 212 | Availability of modern diagnostic services has reduced waiting time |  |  |  |  |  |
| 213 | Digitalizing diagnostic machines has helped to reduce waiting time |  |  |  |  |  |
| 214 | Computerization has helped to reduce registration time resulting in shorter waiting time |  |  |  |  |  |
|  | |  |  |  |  |  |

**SECTION C: OPD SERVICE AREA TIME**

**Instructions: Please indicate/write the time the patient is given the service**

| **S/N** | **Activity** | **Time (hh:mm)** | **Remarks** |
| --- | --- | --- | --- |
| 301 | Time of arrival at the OPD/clinic |  |  |
| 302 | Time at which the queue number is given |  |  |
| 303 | Time Registered in the system |  |  |
| 304 | Time paid (write time indicated from the receipt) |  |  |
| 305 | Time attended at the Triage desk |  |  |
| 306 | Time called to see the Doctor |  |  |
| 307 | Time finished consultation |  |  |
|  |  |  |  |
| **Pharmacy Area** | | | |
| 308 | Time arrival at Pharmacy |  |  |
|  | Time drugs dispensed |  |  |
|  |  |  |  |
| **Laboratory Area** | | | |
| 309  310 | Time arrival at lab reception to get queue number |  |  |
|  | Time Specimen is taken |  |  |
|  |  |  |  |
| **Radiology Area** | | | |
| 310 | Time arrival at the Radiology reception |  |  |
|  | Time investigation is done at Room 4 (X-ray) |  |  |
|  | Time investigation is done at Room 6 (X-ray) |  |  |
|  | Time investigation is done – EMD – Special X-ray |  |  |
|  | Time investigation is done – Ultrasound |  |  |
|  | Time investigation is done – ECHO |  |  |
|  | Time investigation is done – MRI |  |  |
|  | Time investigation is done – CT - SCAN |  |  |
|  |  |  |  |
| 311 | Time of departure from the OPD/Exit time |  |  |

**THE END OF THE QUESTIONS – THANK YOU**

# APPENDIX II: INTERVIEW GUIDE FOR PATIENTS

My name is Manasseh Joel Mwanswila a student of MSc in Health Monitoring and Evaluation. I am conducting a study on ***“OUTCOME EVALUATION OF TECHNICAL STRATEGIES ON REDUCTION OF PATIENT WAITING TIME IN THE OUTPATIENT DEPARTMENT OF KILIMANJARO CHRISTIAN MEDICAL CENTRE - NORTHERN TANZANIA”****.* You are kindly requested to participate in this study by giving your views, opinions and experience. Your participation is important as it will assist in improving care of patients. All information which you are going to give will be confidential and will be used for research only and not for any other purpose. Your participation in this research is entirely voluntary, and you can choose not to answer some of the question, which you do not feel comfortable to answer. After reading the above information with proper explanation given to you and voluntarily you agree to join the study.

**Basic participant information**

| Gender |  |
| --- | --- |
| Age |  |
| Marital status |  |
| Level of education |  |
| Occupation |  |
| Address/Residence |  |

**Technical Strategies**

1. In what way has new Block appointment system reduced waiting time?
2. What is your preference the new block appointment system to the previous appointment system in terms of wait times?
3. How has Migrating from manual to electronic health record proved to minimize prolonged waiting time at the OPD?
4. In what ways has electronic medical records system improved the communication between healthcare providers, which may have resulted in shorter waiting times?
5. How has Extension of clinic days helped to reduce waiting time?
6. How has digitalizing diagnostic machines helped to reduce waiting time?

*Probing hints:* Block appointment system; computerized medical records; Extension of clinic days; Hourly appointment system; Modern diagnostic services

**THE END OF THE QUESTIONS – THAN YOU**

# APPENDIX III: INTERVIEW GUIDE FOR HEALTHCARE PROVIDERS

My name is Manasseh Joel Mwanswila a student of MSc in Health Monitoring and Evaluation. I am conducting a study on ***“OUTCOME EVALUATION OF TECHNICAL STRATEGIES ON REDUCTION OF PATIENT WAITING TIME IN THE OUTPATIENT DEPARTMENT OF KILIMANJARO CHRISTIAN MEDICAL CENTRE - NORTHERN TANZANIA”*** You are kindly requested to participate in this study by giving your views, opinions and experience. Your participation is important as it will assist in improving care of patients. All information which you are going to give will be confidential and will be used for research only and not for any other purpose. Your participation in this research is entirely voluntary, and you can choose not to answer some of the question, which you do not feel comfortable to answer. After reading the above information with proper explanation given to you and voluntarily you agree to join the study.

**Basic participant information**

| Gender |  |
| --- | --- |
| Age |  |
| Marital status |  |
| Level of education |  |
| Occupation |  |

**Technical Strategies**

1. In what way has new Block appointment system reduced waiting time?
2. What is your preference the new block appointment system to the previous appointment system in terms of wait times?
3. How has Migrating from manual to electronic health record proved to minimize prolonged waiting time at the OPD?
4. In what ways has electronic medical records system improved the communication between healthcare providers, which may have resulted in shorter waiting times?
5. How has Extension of clinic days helped to reduce waiting time?
6. How has digitalizing diagnostic machines helped to reduce waiting time?

*Probing hints:* Block appointment system; computerized medical records; Extension of clinic days; Hourly appointment system; Modern diagnostic services

**THE END OF THE QUESTIONS – THAN YOU**

**DODOSO KWA WAGONJWA**

Jina langu ni **Manasseh Joel Mwanswila,** mwanafunzi wa Uzamili (MSc) katika Ufuatiliaji na Tathmini ya Afya katika Chuo Kikuu cha Mzumbe. Ningependa kukuuliza maswali machache kuhusu mikakati ya kuingilia kati iliyotekelezwa na hospitali ili kupunguza muda wa kusubiri huduma za afya za wagonjwa wa nje katika Hospitali ya KCMC.

**Kichwa cha utafiti**: Ninafanya utafiti juu ya “***TATHMINI YA MATOKEO YA MIKAKATI YA KIUFUNDI KATIKA KUPUNGUZA MUDA WA KUSUBIRI WAGONJWA WA NJE KATIKA IDARA YA WAGONJWA WA NJE YA HOSPITALI YA KILIMANJARO CHRISTIAN MEDICAL CENTRE - KASKAZINI MWA TANZANIA”****.* Umechaguliwa kama mshiriki katika utafiti huu kutoa maoni yako, maoni na uzoefu wako na ushiriki wako ni muhimu kwani utasaidia kuboresha huduma kwa wagonjwa. Matokeo ya utafiti huu yatatumika kutoa taarifa kama msingi hasa katika eneo la utafiti kwa watafiti ili kushughulikia tatizo hilo.

**Usiri:** Taarifa zote zitakazopatikana kutoka kwako wakati wa utafiti zitahifadhiwa kwa usiri kabisa, ni mtafiti mkuu pekee atakayepata ufikiaji wake na zitatumika kwa utafiti pekee na sio kwa madhumuni mengine yoyote. Jina lako halitatajwa katika utafiti au katika ripoti za matokeo. Tafadhali jisikie huru kushiriki.

**Ridhia ya hiari:** Ushiriki wako katika utafiti huu ni wa hiari kabisa. Tafadhali jisikie huru kuuliza maswali yoyote kabla au baada ya mahojiano na unaweza kuchagua kutokujibu baadhi ya maswali ambayo hujisikii vizuri kuyajibu. Pia, uko huru kukataa kushiriki katika utafiti wakati wowote na hutakutana na matokeo yoyote kama utaamua hivyo.

Je, uko tayari kushiriki? NDIO HAPANA

Ikiwa NDIO: Sahihi ……………………………..Tarehe…………………………………………

Jina la Mtafiti ………………………………………………………………………………………

Sahihi ……………………………………………. Tarehe ………………………………………..

**Asante kwa ushirikiano wako.**

**DODOSO KWA WAGONJWA (TOLEO LA KISWAHILI)**

**Nambari ya Usajili ya Hospitali ……………………… Tarehe ………………………………**

**Jina la Kliniki …………………………………Siku ya wiki …………………………………..**

**Maelekezo:** Tafadhali weka alama ya tiki kwenye jibu sahihi zaidi **(√)**

**SEHEMU A: TAARIFA ZA KIJAMII**

1. Jinsia (weka tiki kwenye kisanduku)
2. Mwanaume ( )
3. Mwanamke ( )
4. Umri wako ni ?..............................................
5. Hali yako ya ndoa ni ipi?
6. Umeoa/Umeolewa ( )
7. Sijaoa/Sijaolewa ( )
8. Umetalikiana/Umetengana ( )
9. Mjane/Mgane ( )
10. Kiwango chako cha elimu ni kipi? Tafadhali weka tiki kwenye kisanduku.
11. Sijaenda shule ( )
12. Shule ya Msingi ( )
13. Shule ya Sekondari ( )
14. Chuo (cheti au Astashahada) ( )
15. Chuo Kikuu (Shahada/Uzamili/Uzamivu) ( )
16. Kazi yako ni ipi?
17. Mkulima ( )
18. Mfanyabiashara ( )
19. Mwajiriwa ( )
20. Umejiajiri ( )
21. Mwanafunzi ( )
22. Makazi
23. Ndani ya eneo linalohudumiwa [Kilimanjaro Arusha Manyara Tanga Singida]( )
24. Nje ya eneo linalohudumiwa ( )
25. Njia ya malipo
26. Fedha taslimu ( )
27. Bima ( )
28. Msamaha ( )
29. Tangu lini umeanza kuhudhuria kliniki ya KCMC
30. 2018 na chini ( )
31. 2019 ( )
32. 2020 ( )
33. 2021 ( )
34. 2022 ( )
35. 2023 ( )

**SEHEMU B:** Tafadhali onyesha kiwango chako cha Kuridhishwa kwa kauli zifuatazo kwa kuweka alama ya **tiki (√)** kwenye mojawapo ya chaguzi. **UFUNGUO**: **1 = SIKUBALIANI KABISA; 2 = SIKUBALIANI; 3 = NEUTRAL** **(nakubaliana wala sikubaliani); 4 = NAKUBALI; 5 = NAKUBALI KABISA**

| **Tabia** | |  |  |  |  |  |
| --- | --- | --- | --- | --- | --- | --- |
| **Vipengele cha kiufundi** | | **1** | **2** | **3** | **4** | **5** |
| 201 | Mfumo mpya wa miadi ya Block umepunguza muda wa kusubiri |  |  |  |  |  |
| 202 | Ninapendelea mfumo wa miadi ya Block kuliko mfumo wa awali kwa suala la muda wa kusubiri |  |  |  |  |  |
| 203 | Kuanzishwa kwa miadi ya kila saa kumeweza kupunguza muda wa kusubiri |  |  |  |  |  |
| 204 | Kusambaza ujio wa wagonjwa kwa usawa zaidi siku nzima husaidia kupunguza muda wa kusubiri |  |  |  |  |  |
| 205 | Upungufu wa kutumia nyaraka za karatasi unashindwa na rekodi za matibabu za kielektroniki, na kusababisha muda mfupi wa kusubiri |  |  |  |  |  |
| 206 | Mfumo wa rekodi za matibabu za kielektroniki umeboresha mawasiliano kati ya watoa huduma za afya, hivyo kusababisha muda mfupi wa kusubiri |  |  |  |  |  |
| 207 | Rahisi kupata taarifa za mgonjwa kwa mfumo wa rekodi za matibabu za kielektroniki, na hivyo kusababisha muda mfupi wa kusubiri |  |  |  |  |  |
| 208 | Kutumia EMR muda unaohitajika kusubiri nyaraka unapungua, hivyo muda wa kusubiri kwa mgonjwa unapungua |  |  |  |  |  |
| 209 | Kuhama kutoka rekodi za mwongozo hadi za kielektroniki kumethibitisha kupunguza muda mrefu wa kusubiri katika OPD |  |  |  |  |  |
| 210 | Kuongezwa kwa siku za kliniki kumeongeza muda wa kusubiri |  |  |  |  |  |
| 211 | Kuongezwa kwa siku za kliniki kimenipa chaguo zaidi za siku za kliniki zinazolingana na ratiba yangu |  |  |  |  |  |
| 212 | Uwepo wa huduma za kisasa za uchunguzi umepunguza muda wa kusubiri |  |  |  |  |  |
| 213 | Kidigitali mashine za uchunguzi kumesaidia kupunguza muda wa kusubiri |  |  |  |  |  |
| 214 | Ukompyuta umesaidia kupunguza muda wa usajili na hivyo kusababisha muda mfupi wa kusubiri |  |  |  |  |  |
|  | |  |  |  |  |  |

**SEHEMU C: MUDA UNAOTUMIKA ENEO LA HUDUMA ZA WAGONJWA WA NJE (OPD)**

**Maelekezo: Tafadhali onyesha/andika muda ambao mgonjwa amepewa huduma**

| **S/N** | **Shughuli** | | **Wakati/Muda (Saa:dakika)** | **Maoni** |
| --- | --- | --- | --- | --- |
| 301 | Wakati wa kufika katika OPD/kliniki | |  |  |
| 302 | Wakati namba ya foleni inapopatikana | |  |  |
| 303 | Wakati wa kusajiliwa katika mfumo | |  |  |
| 304 | Wakati wa kulipia (andika muda uliyoonyeshwa kwenye risiti) | |  |  |
| 305 | Wakati wa kuhudumiwa katika dawati la vipimo vya awali(Triage) | |  |  |
| 306 | Wakati wa kuitwa kumuona Daktari | |  |  |
| 307 | Wakati wa kumaliza ushauri | |  |  |
|  |  | |  |  |
| **Eneo la Dawa** | | | | |
| 308 | Wakati wa kufika katika Duka la Dawa | |  |  |
|  | Wakati wa kupewa dawa | |  |  |
|  |  | |  |  |
| **Eneo la Maabara** | | | | |
| 309  310 | Wakati wa kufika katika mapokezi ya maabara ili kupata namba ya foleni |  | |  |
|  | Wakati wa Sampuli inachukuliwa |  | |  |
|  |  |  | |  |
| **Eneo la Radiolojia** | | | | |
| 310 | Wakati wa kufika katika mapokezi ya Radiolojia |  | |  |
|  | Wakati wa kufanyiwa uchunguzi katika Chumba 4 (X-ray) |  | |  |
|  | Wakati wa kufanyiwa uchunguzi katika Chumba 6 (X-ray) |  | |  |
|  | Wakati wa kufanyiwa uchunguzi – EMD – X-ray Maalum |  | |  |
|  | Wakati wa kufanyiwa uchunguzi – Ultrasound |  | |  |
|  | Wakati wa kufanyiwa uchunguzi – ECHO |  | |  |
|  | Wakati wa kufanyiwa uchunguzi – MRI |  | |  |
|  | Wakati wa kufanyiwa uchunguzi – CT - SCAN |  | |  |
|  |  |  | |  |
| 311 | Wakati wa kuondoka kutoka OPD/Muda wa kutoka |  | |  |

**MWISHO WA MASWALI – ASANTE**

**MWONGOZO WA MAHOJIANO KWA WAGONJWA**

Jina langu ni Manasseh Joel Mwanswila, mwanafunzi wa Uzamili (MSc) katika Ufuatiliaji na Tathmini ya Afya. Ninafanya utafiti juu ya **"*TATHMINI YA MATOKEO YA MIKAKATI YA KIUFUNDI KATIKA KUPUNGUZA MUDA WA KUSUBIRI WAGONJWA WA NJE KATIKA IDARA YA WAGONJWA WA NJE YA HOSPITALI YA KILIMANJARO CHRISTIAN MEDICAL CENTRE - KASKAZINI MWA TANZANIA*"**. Unakaribishwa kwa heshima kushiriki katika utafiti huu kwa kutoa maoni yako, maoni na uzoefu wako. Ushiriki wako ni muhimu kwani utasaidia kuboresha huduma kwa wagonjwa. Taarifa zote utakazotoa zitakuwa za siri na zitatumika kwa utafiti pekee na sio kwa madhumuni mengine yoyote. Ushiriki wako katika utafiti huu ni wa hiari kabisa na unaweza kuchagua kutokujibu baadhi ya maswali ambayo hujisikii vizuri kuyajibu. Baada ya kusoma taarifa zilizo hapo juu na maelezo sahihi yaliyotolewa kwako na kwa hiari unakubali kujiunga na utafiti.

**Taarifa za Msingi za Mshiriki**

| Jinsia |  |
| --- | --- |
| Umri |  |
| Hali ya ndoa |  |
| Kiwango cha elimu |  |
| Aina ya kazi unayofanya |  |

**Mikakati ya Kiufundi**

1. Ni kwa namna gani mfumo mpya wa miadi ya Block umepunguza muda wa kusubiri?
2. Ni upi upendeleo wako kati ya mfumo mpya wa miadi ya Block na mfumo wa awali kwa suala la muda wa kusubiri?
3. Kuhama kutoka rekodi za mwongozo hadi za kielektroniki kumethibitishaje kupunguza muda mrefu wa kusubiri katika OPD?
4. Ni kwa namna gani mfumo wa rekodi za matibabu za kielektroniki umeboresha mawasiliano kati ya watoa huduma za afya, hivyo kusababisha muda mfupi wa kusubiri?
5. Kuongezwa kwa siku za kliniki kumesaidaje kupunguza muda wa kusubiri?
6. Kidigitali mashine za uchunguzi kumesaidaje kupunguza muda wa kusubiri?

**Vidokezo vya Kuchochea Majadiliano**

Mfumo wa miadi ya Block,Rekodi za matibabu zilizokompyuta,Kuongezwa kwa siku za kliniki,Mfumo wa miadi ya kila saa,Huduma za uchunguzi wa kisasa

**MWISHO WA MASWALI – ASANTE**

**MWONGOZO WA MAHOJIANO KWA WATOA HUDUMA**

Jina langu ni Manasseh Joel Mwanswila, mwanafunzi wa Uzamili (MSc) katika Ufuatiliaji na Tathmini ya Afya. Ninafanya utafiti juu ya “***TATHMINI YA MATOKEO YA MIKAKATI YA KIUFUNDI KATIKA KUPUNGUZA MUDA WA KUSUBIRI WAGONJWA WA NJE KATIKA IDARA YA WAGONJWA WA NJE YA HOSPITALI YA KILIMANJARO CHRISTIAN MEDICAL CENTRE - KASKAZINI MWA TANZANIA”***. Unakaribishwa kwa heshima kushiriki katika utafiti huu kwa kutoa maoni yako, maoni na uzoefu wako. Ushiriki wako ni muhimu kwani utasaidia kuboresha huduma kwa wagonjwa. Taarifa zote utakazotoa zitakuwa za siri na zitatumika kwa utafiti pekee na sio kwa madhumuni mengine yoyote. Ushiriki wako katika utafiti huu ni wa hiari kabisa na unaweza kuchagua kutokujibu baadhi ya maswali ambayo hujisikii vizuri kuyajibu. Baada ya kusoma taarifa zilizo hapo juu na maelezo sahihi yaliyotolewa kwako na kwa hiari unakubali kujiunga na utafiti.

**Taarifa za Msingi za Mshiriki**

| Jinsia |  |
| --- | --- |
| Umri |  |
| Hali ya ndoa |  |
| Kiwango cha elimu |  |
| Aina ya kazi unayofanya |  |

**Mikakati ya Kiufundi**

1. Ni kwa namna gani mfumo mpya wa miadi ya Block umepunguza muda wa kusubiri?
2. Ni upi upendeleo wako kati ya mfumo mpya wa miadi ya Block na mfumo wa awali kwa suala la muda wa kusubiri?
3. Kuhama kutoka rekodi za mwongozo hadi za kielektroniki kumethibitishaje kupunguza muda mrefu wa kusubiri katika OPD?
4. Ni kwa namna gani mfumo wa rekodi za matibabu za kielektroniki umeboresha mawasiliano kati ya watoa huduma za afya, hivyo kusababisha muda mfupi wa kusubiri?
5. Kuongezwa kwa siku za kliniki kumesaidaje kupunguza muda wa kusubiri?
6. Kidigitali mashine za uchunguzi kumesaidaje kupunguza muda wa kusubiri?

**Vidokezo vya Kuchochea Majadiliano**

Mfumo wa miadi ya Block,Rekodi za matibabu zilizokompyuta, Kuongezwa kwa siku za kliniki, Mfumo wa miadi ya kila saa, Huduma za uchunguzi wa kisasa

**MWISHO WA MASWALI – ASANTE**
